# Supplementary material for: The RSL3 Induction of KLK Lung Adenocarcinoma Cell Ferroptosis by Inhibition of USP11 Activity and the NRF2-GSH Axis
Source: Cancers (Basel). 2022 Oct 25;14(21):5233. doi: 10.3390/cancers14215233 (PMC9658295; doi:10.3390/cancers14215233)
Supplement: Supplementary file 1 [file cancers-14-05233-s001.zip › cancers-1967467-SI.pdf]

Supplemental materials and methods

Cell culture

Human LUAD cell lines H23 and H1299 were originally obtained from the American Type Culture Collection (ATCC; Manassas, VA, USA) and grown in Roswell Park Memorial Institute medium-1640 (RPMI-1640; Gibco) supplemented with 10% FBS (Gibco) and 1% penicillin/streptomycin (Sigma) in a humidified incubator with 5% CO2 at 37°C.

Detection Fe2+ level

Cells were first seeded into fluorescent petri dishes and grown overnight at 37°C in a 5% CO2 incubator. To detect Fe2+ level, cells were washed with the serum-free medium three times and added with the growth medium containing different concentrations of RSL3 (Table 1) and incubated in a 5% CO2 incubator at 37°C for 24 h. After that, cells were washed with the serum-free medium three times and then added with 1 μM FerroOrange and incubated at 37°C in a 5% CO2 incubator for 30 min. The cells were afterward, reviewed and photographed under a fluorescence microscope.

Transmission electron microscopy

A549 and H2122 cells were treated with RSL3 (8 μM) for 8 h. The suspended cells were collected by centrifugation at 1,000 rpm for 5 min and then fixed in 10 ml of 2.5% glutaraldehyde/phosphate buffer for 2 h at the room temperature. The samples were then washed with PBS and further fixed with 1% osmic acid. After that, the samples were dehydrated, embedded, solidified, sectioned, and stained for observation under transmission electron microscope (jem-1230) at 80 KV.

Cystine uptake assay

The cystine uptake ability was detected by cystine uptake kit (DOJINDO). In brief, cells were seeded into 96-well plates overnight and then washed three times with 200 μl cystine and serum-free medium that was preheat at 37°C. The Cystine and serum-free medium containing DMSO, RSL3 (8 μM) or Erastin (20 μM) were added into cell culture and incubated at 37°C in 5 % CO2 incubator for 5 min. After removing the supernatant, 200 μl of cystine and serum-free cystine containing cystine analogue medium was added into cells. DMSO, RSL3 (8 μM) or Erastin (20 μM) were subsequently added, respectively, into cells and cells were incubated for 2h at 37°C in 5% CO2 incubator. After that, cells were washed three times with 200 μl ice-cold PBS and added with 50 μl methanol; 200 μl of the working solution containing the probe was added to each well and incubate the cells at 37°C in 5% CO2 incubator for 30 min. The fluorescence (λEX = 490 nm and λEM = 535 nm) was detected with a luciferase microplate reader (Infinite M200 PRO, TECAN). The fluorescence intensity produced by cystine analog absorbed by cells was then calculated.

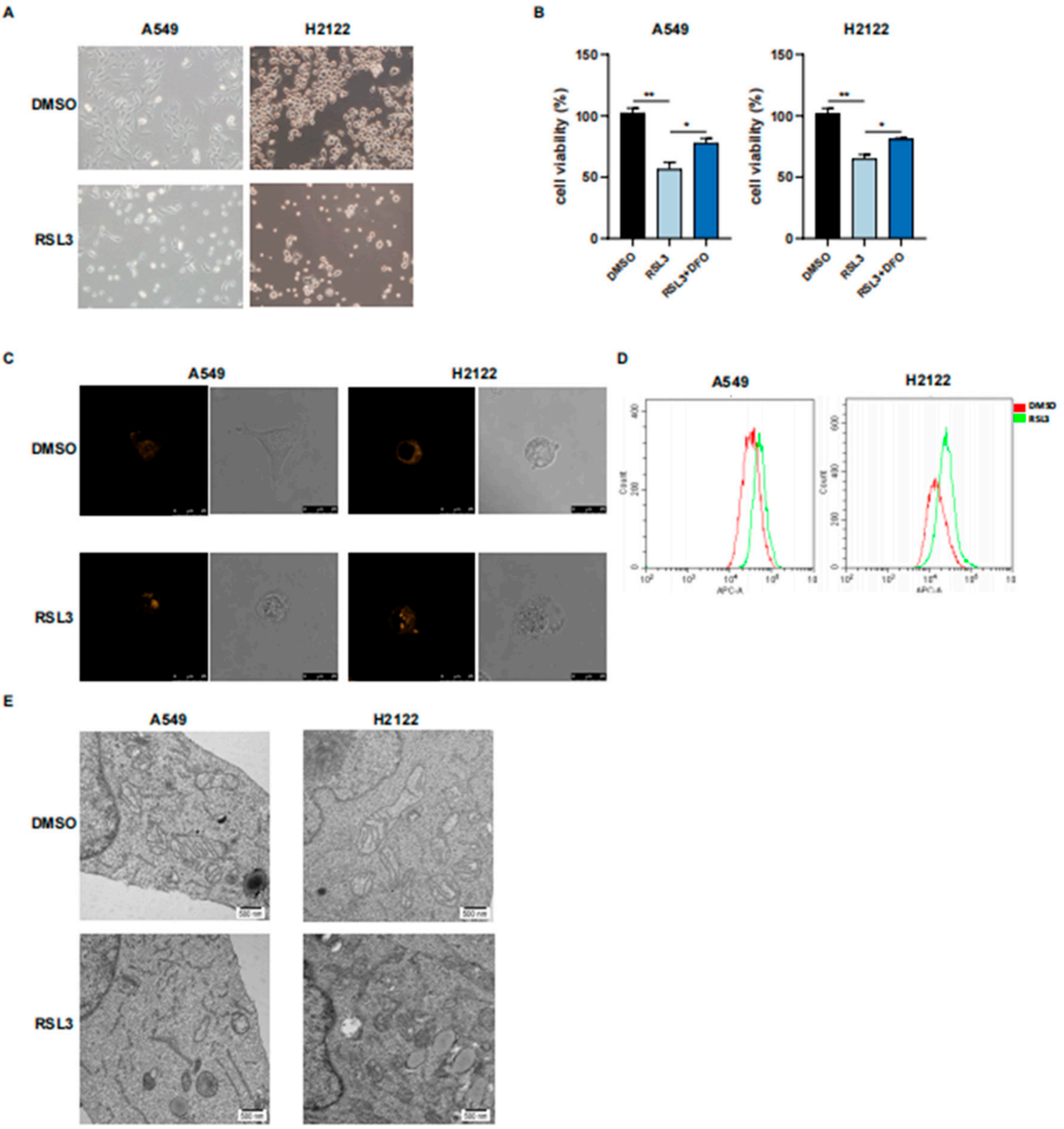

Figure S1. RSL3 induction of KLK LUAD cell ferroptosis. (A) Cell viability assay under the phase contrast microscope. Indicated cells were treated with dimethyl sulfoxide (DMSO) or RSL3 (8 μM) for 24 h and then observed under the phase contrast microscope. The representative images of ferroptotic cell death induced by RSL3 was shown. (B) Summarized data on cell viability CCK-8 assay. Indicated cells were pretreated with DFO (100 μM) for 4 h, then treated with dimethyl sulfoxide (DMSO) or RSL3 (8 μM) for 24 h and subjected to the CCK-8 assay. (C) FerrOrange assay. Indicated cells were treated with dimethyl sulfoxide (DMSO) or RSL3 (8 μM) for 24 h and then subjected to the FerrOrange assay. The representative Fe2+ images in indicated cells were shown. (D) Lipid peroxidation and flow cytometric FACS assay. Indicated cells were treated with dimethyl sulfoxide (DMSO) or RSL3 (8 μM) for 24 h and then subjected to the FACS assay. (E) Transmission electron microscopy. Indicated cells were treated with DMSO or RSL3 (8 μM) for 24 h and the mitochondria morphology and structure were observed under the TEM. The data were expressed as mean ± SD. **P* < 0.05 and ***P* < 0.01 versus the control group.

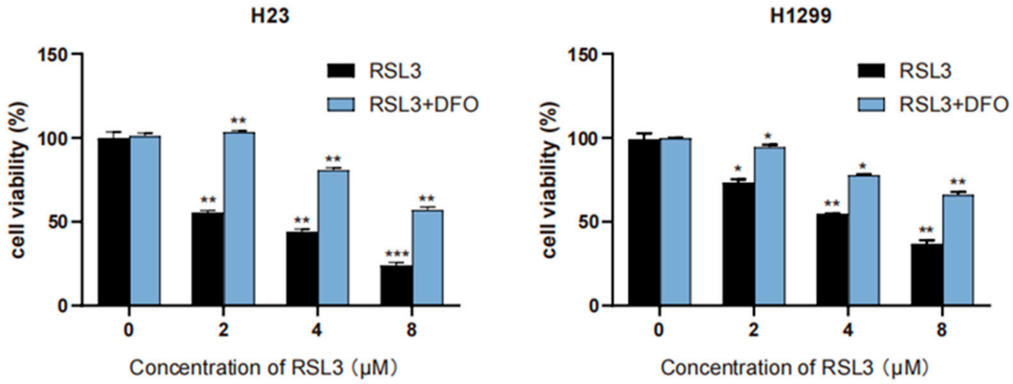

Figure S2. RSL3 (2-8 μM) induction of H23 and H1299 cell death in a dose-dependent manner that was rescued by the DFO treatment (100 μM). The data were expressed as mean ± SD. *$P < 0.05$ and **$P < 0.01$ versus the control group.

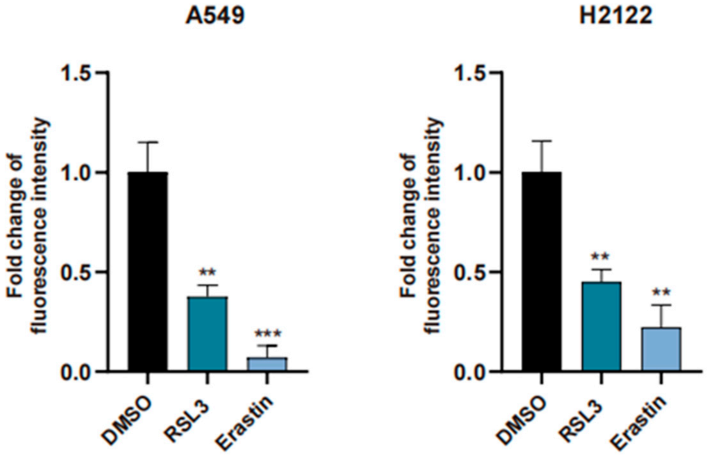

Figure S3. RSL3 reduction of the cystine uptake in KLK LUAD cells as Erastin did. The data were expressed as mean ± SD. **$P < 0.01$ and ***$P < 0.001$ versus the control group.

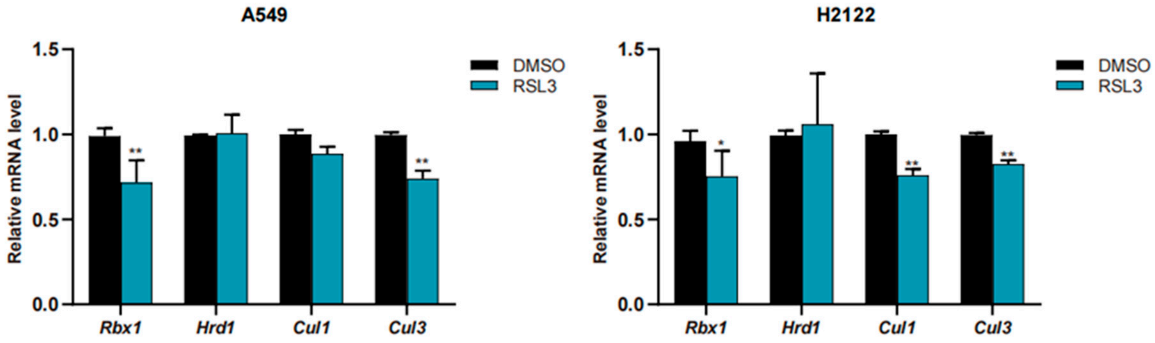

Figure S4. Effects of RSL3 on expression of E3 ligases of NRF2 in KLK LUAD cells. Indicated cells were treated with dimethyl sulfoxide (DMSO) or RSL3 (8 μM) for 12 h and then subjected to qRT-PCR analysis of indicated E3 ligase mRNA. *$P < 0.05$ and **$P < 0.01$ versus the control group.

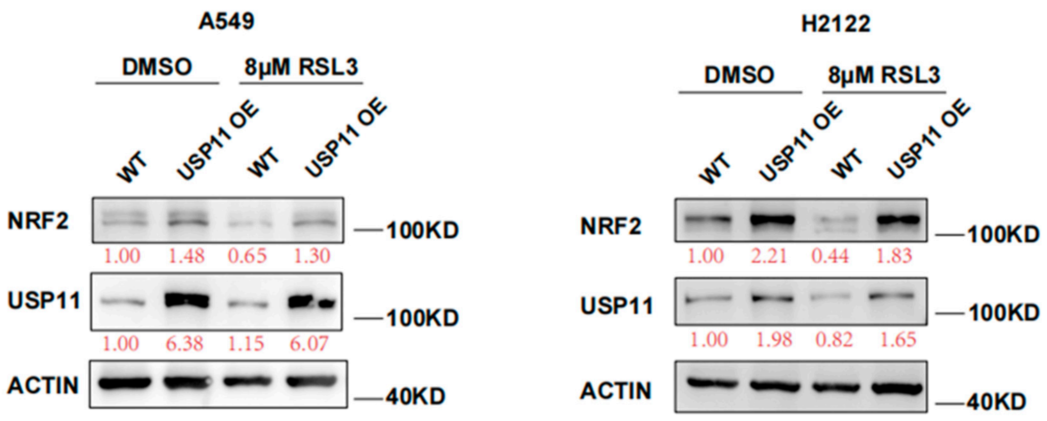

Figure S5. Construction of USP11 overexpressed A549 and H2122 cells. Indicated cells were treated with dimethyl sulfoxide (DMSO) or RSL3 (8 μM) for 12 h and then subjected to Western blotting analysis of USP11 and NRF2 proteins.
